# Experimental Investigations of Using Aluminum Oxide (Al_2_O_3_) and Nano-Graphene Powder in the Electrical Discharge Machining of Titanium Alloy

**DOI:** 10.3390/mi14122247

**Published:** 2023-12-16

**Authors:** Rakesh Chaudhari, Sakshum Khanna, Vivek K. Patel, Jay Vora, Soraya Plaza, Luis Norberto López de Lacalle

**Affiliations:** 1 Department of Mechanical Engineering, School of Technology, Pandit Deendayal Energy University, Gandhinagar 382007, India; rakesh.chaudhari@sot.pdpu.ac.in (R.C.); vivekp@sot.pdpu.ac.in (V.K.P.); 2 School of Technology, Pandit Deendayal Energy University, Gandhinagar 382007, India; sakshum.khanna@gmail.com; 3 Department of Mechanical Engineering, University of the Basque Country, Escuela Superior de Ingenieros Alameda de Urquijo s/n, 48013 Bilbao, Spain; soraya.plaza@ehu.eus

**Keywords:** EDM, Ti6Al4V, TLBO algorithm, aluminum oxide (Al_2_O_3_) nanopowder, nano-graphene

## Abstract

In the present study, a comprehensive parametric analysis was carried out using the electrical discharge machining of Ti6Al4V, using pulse-on time, current, and pulse-off time as input factors with output measures of surface roughness and material removal rate. The present study also used two different nanopowders, namely alumina and nano-graphene, to analyze their effect on output measures and surface defects. All the experimental runs were performed using Taguchi’s array at three levels. Analysis of variance was employed to study the statistical significance. Empirical relations were generated through Minitab. The regression model term was observed to be significant for both the output responses, which suggested that the generated regressions were adequate. Among the input factors, pulse-off time and current were found to have a vital role in the change in material removal rate, while pulse-on time was observed as a vital input parameter. For surface quality, pulse-on time and pulse-off time were recognized to be influential parameters, while current was observed to be an insignificant factor. Teaching–learning-based optimization was used for the optimization of output responses. The influence of alumina and nano-graphene powder was investigated at optimal process parameters. The machining performance was significantly improved by using both powder-mixed electrical discharge machining as compared to the conventional method. Due to the higher conductivity of nano-graphene powder, it showed a larger improvement as compared to alumina powder. Lastly, scanning electron microscopy was operated to investigate the impact of alumina and graphene powder on surface morphology. The machined surface obtained for the conventional process depicted more surface defects than the powder-mixed process, which is key in aeronautical applications.

## 1. Introduction

Owing to excellent properties like higher resistance to corrosion, light weight, and biocompatibility, Ti6Al4V is one of the most used titanium alloys in various sectors [1,2]. Thus, Ti6Al4V is largely used in various automotive parts, aerospace components, biomedical devices, and several other sectors, such as the oil and gas, marine, energy, and infrastructure sectors [3,4,5]. Along with the numerous advantages of Ti6Al4V, their higher strength and poor thermal conductivity impose lots of challenges through conventional machining techniques such as excessive tool wear, unsuitable chip breakage, and poor surface finish [6,7]. To overcome these limitations, nonconventional machining techniques can be effectively used for machining hard materials [8,9,10]. Ti6Al4V was used in various advanced manufacturing techniques like 3D printing, including laser powder bed fusion, electron beam melting, directed energy deposition, and nonconventional machining processes [11,12,13]. Electrical discharge machining (EDM) is a type of nontraditional machining technique that can be effectively used to produce complex shape parts with better surface finish and accuracy [14,15,16]. EDM erodes the workpiece particles by forming regulated electric sparks among the work and tool material in the presence of suitable dielectric fluid [17,18]. The tool and work material need to be electrically conductive for machining through EDM [19,20]. The EDM process consists of several input factors that require balance to attain the desired outcome [21,22]. This requires a systematic experimental approach. Taguchi’s design provides a systematic experimental design for various input variables, with numerous features, like decreasing the number of trials, thereby saving cost and time, and an empirical relationship between the machining variables and output measures [23,24]. During the machining, higher productivity along with a better surface finish is always desirable [25]. Thus, the current work aims to maximize the material removal rate (MRR) and reduction in surface roughness (SR).

Several studies were conducted to optimize the output responses of the EDM process for titanium alloys. Dikshit et al. [26] preferred the EDM method to study the surface characteristics of Ti6Al4V alloy by considering the process pulse-on time (T_on_), current (*I*), and pulse-off time (T_off_) as EDM variables. The obtained results have shown that *I* was detected as the largest influencing factor for both SR and MRR. Pinargote et al. [27] used a wire-EDM process of spark plasma sintered SiC-TiB_2_-TiC ceramic composite to minimize recast layer thickness and SR. They utilized the combined approach of Taguchi and grey relational analysis to determine optimal variables. Another study conducted by Devarasiddappa et al. [28] preferred the EDM process to optimize the SR response of Ti6Al4V. They employed Taguchi’s method to design experiments by considering T_on_, wire speed, *I*, and T_off_ as machining parameters. Their employed method of teaching–learning-based optimization (TLBO) has shown improvement in SR by 2.65%. T_on_ and *I* were observed to have a vital impact on SR response, with contributions of 44.06%, and 28.69%, respectively, followed by T_off_ with 15.8% and wire speed of 7.47%. Lower values of T_on_, and *I* revealed a defect-free surface obtained through scanning electron microscopy (SEM). In a study performed by Vora et al. [29], Taguchi’s design was used during the wire-EDM process of Ti6Al4V alloy. Their finding revealed that T_on_ and *I* had the most influencing factors for MRR and SR, respectively. A parametric study conducted by Guo et al. [30] analyzed the effect of EDM factors on SR and surface integrity of Ti6Al4V alloy using Taguchi’s L16 array. Pareto points were derived from the nondominated sorting genetic algorithm to predict the solutions. In another study carried out by Verma and Sajeevan [31], a die-sinking EDM process was preferred to optimize the performance of Ti6Al4V. They revealed that the EDM process provides poor surface integrity while machining Ti alloys. They analyzed the machined surfaces through SEM and observed the larger presence of recast layer formation and the development of microcracks. Thus, based on the recent work, T_on_, T_off_, and current were observed to have a larger significance on output characteristics of the EDM process.

It is essential to reduce surface defects with simultaneous improvement in machining rate [32,33]. In addition to optimizing the process variables, a new approach needs to be implemented which should enhance MRR and reduce SR along with the improvement in surface characteristics. The inclusion of nanopowders in dielectric fluid with the proper amount can significantly enhance the machining features [34,35,36]. The addition of nanopowders enlarges the thermal conductivity, increases the discharge gap, decreases the breakdown strength, and enhances the spark difference [37,38,39]. In past studies, several nanopowder concentrations were used by the researchers to enhance the machining outcomes [40]. A comprehensive study shown by Taherkhani et al. [41] used microalumina (µ-Al_2_O_3_) powder to improve the EDM machining performance of Ti6Al4V alloy. The surface defects were largely eliminated due to the presence of alumina powder. The addition of alumina powder lowered the surface crack density and formed a uniform surface. Chaudhari et al. [42] analyzed the impact of alumina powder amount on MRR, tool wear rate (TWR), and SR through the die-sinking EMD process of Nitinol. The amounts of alumina powder, T_off_, and T_on_ were detected as vital input factors to have a significant impact on all output measures. Alumina powder was the highest contributing factor for the enhancement in MRR. SR and TWR were also decreased with the addition of alumina powder. SEM analysis has revealed substantial improvement in surface morphology owing to the suspended nanoparticles. Chaudhari et al. [43] studied the influence of nano-graphene powder on the WEDM process. The suspended nano-graphene powder formed uniform sparking and debris flushing, which reduced SR and improved MRR owing to their high erosion. SR and MRR were found to be increased by 9.35% and 24.01%, respectively, with PMEDM at 1 g/L. SEM analysis revealed the improvement in surface morphology with reduced microcracks and other defects. Vora et al. [44] investigated the effect of nano-graphene powder at various concentrations on the die-sinking EDM of shape memory alloy. Along with nano-graphene PC, T_on_, *I*, and T_off_ were elected as input factors. Taguchi’s L9 was preferred to perform trials. The finding observed that the use of nano-graphene particles showed substantial improvement in MRR by 75.18%. Additionally, the inclusion of nano-graphene powder also reduced SR and dimensional deviation. Surface defects were largely reduced due to the addition of nano-graphene powder. Ishfaq et al. [45] used Taguchi’s L18 design to investigate the impact of nano-graphene on the EDM-machined surface of Ti6Al4V. The experimental finding showed an improvement in surface quality for graphene-mixed dielectric fluid.

Limited work has been reported on the EDM of Ti6Al4V using a nanopowder-mixed dielectric. Surface damage is a clear limitation in blade and aero-engine component machining, including deep holes and narrow slots. The present study used two different nanopowders, namely alumina and nano-graphene, to analyze the effect on SR, MRR, and surface morphology. In the current work, T_on_, *I*, and T_off_ were considered as input factors with output measures of SR and MRR of Ti6Al4V alloy. Empirical relations were generated through Minitab and optimized through the teaching–learning-based optimization (TLBO) algorithm. ANOVA was employed to study the statistical significance. Lastly, SEM was operated to investigate the impact of alumina and graphene powder on surface morphology.

## 2. Materials and Methods

### 2.1. Synthesis of Nanopowders

#### 2.1.1. Aluminum Oxide (Al_2_O_3_) Nanopowder

**Figure 1:**
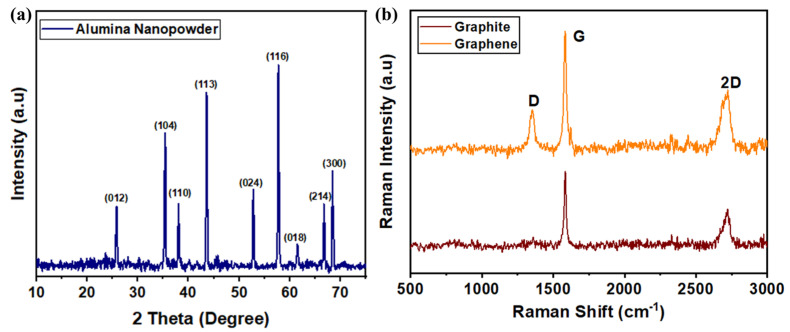
(**a**) XRD profile of alumina [42], (**b**) Raman spectrum of nano-graphene [44].

We utilized a hydrothermal synthesis approach to generate aluminum oxide (Al_2_O_3_) nanopowder without the preliminary purification of chemical reagents [42] A hydrothermal synthesis approach was employed without the prior purification of chemical reagents to produce aluminum oxide (Al_2_O_3_) nanopowder. The key reagents utilized in this process encompassed citric acid, aluminum nitrate nanohydrate, triethanolamine, and ethylene glycol. Throughout the experimental procedures, we maintained a commitment to the use of ultrapure water with an impressive resistivity of 18.2 MΩ-cm to ensure the highest level of precision and accuracy. In a prototypical synthesis procedure, the process was initiated by dissolving aluminum nitrate nanohydrate in deionized water. Employing a medium-speed stirrer, we diligently mixed the components to attain a homogeneous blend. The subsequent step involved the gradual addition of triethanolamine into the mixture, carefully introduced drop by drop. After a period of 40 min, during which the mixture was subjected to stirring at a controlled temperature of 75 °C, citric acid was introduced to the solution. The incorporation of citric acid elicited a noticeable transformation in the coloration of the sols. Continuing with the synthesis process, the sols were heated for a duration of 90 min, maintaining the temperature at 150 °C. This controlled thermal treatment resulted in the sols transforming into highly viscous gels. To further progress towards the desired Al_2_O_3_ nanopowder, the solution was subjected to a final thermal treatment at a temperature of 1200 °C. This heat treatment was sustained for a duration of three hours, effectively facilitating the complete drying process. The culmination of this meticulous procedure yielded the desired Al_2_O_3_ nanopowder, which could subsequently be utilized for a myriad of applications. The average size of the alumina nanopowder was observed to be ~110 nm, which was near to our previously reported work [42]. X-ray diffraction spectroscopy (XRD) was used to confirm the structural formation of alumina. The pattern (Figure 1a) showed peaks at different 2θ values corresponding to the hexagonal structure of α-Al_2_O_3_ (JCPDS No 46-1212), confirming its formation [46].

#### 2.1.2. Nano-Graphene Nanopowder

To produce nano-graphene sheets, an ultrasonication method was used, where 5 g of natural graphite was mixed with 1,2-dichlorobenzene (DCB) within a 500 mL flask. This mixture was then portioned into 10 mL containers and subjected to ultrasonication for a duration of 10 h. To ensure the prevention of water overheating and evaporation during this process, we diligently maintained and altered the water bath as needed. Following the ultrasonication phase, the resultant sample was left undisturbed for 48 h, during which a noticeable grey dispersion emerged. To separate the graphene sheets from any unreacted graphite and achieve a more refined product, the colloidal dispersion was centrifuged at 5000 revolutions per minute (rpm) for a duration of 15 min. As a result, the heavy lumps of unreacted graphite settled at the bottom, leaving behind the desired graphene sheets in the supernatant. To further enhance the quality and uniformity of the graphene dispersion, it was carefully transferred to a separate vial and dispersed in an ethanol solution. This critical step was repeated 3 to 4 times to optimize the dispersion’s homogeneity. In the final stages of the process, we subjected the centrifuged graphene sample to filtration and drying within a vacuum furnace. This step was crucial for removing any excess ethanol and DCB, ensuring the purity of the graphene sheets. One remarkable aspect of this method was its ability to maintain the dispersion of sonicated graphene sheets for an extended period, even after several months. Raman spectroscopy confirmed the presence of graphene nanopowder (Figure 1b). Its characteristic 2D band exhibited a red shift compared to natural graphite, indicating the successful production of few-layered graphene sheets. Minor defects identified by the D band likely originated from the exfoliation process [47].

### 2.2. Experimental Conditions

**Figure 2:**
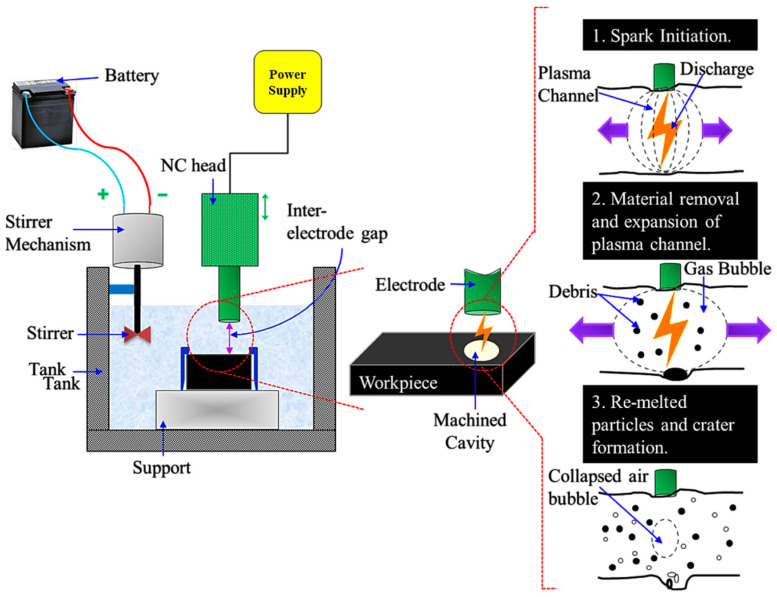
Schematic of die-sinking EDM process [48].

The present investigation used Sparkonix-made die-sinking EDM (Sparkonix, Pune, India) to perform the experimental runs. Figure 2 depicts the schematic and basic principle of the die-sinking EDM process. In the present study, EDM oil was used as a dielectric fluid. Ti6Al4V alloy was utilized as work material, and brass as the tool electrode with 10 mm diameter. The key elements of the work material consisted of 6% of Al, 4% of V, and Ti as balance. T_on_, *I*, and T_off_ were considered as input factors with output measures of SR and MRR. Later, aluminum oxide (Al_2_O_3_) nanopowder, and nano-graphene powder were used at 1 g/L amount. During the experimentations, 2 mm of cutting depth with a constant spark gap of 0.01 mm was kept. The experimental runs were performed as per Taguchi’s design at 3 levels with nine experimental trials. Table 1 depicts the input factors at various levels and other experimental conditions. Empirical relations were generated through Minitab v17 software. ANOVA was employed to study the statistical significance of machining factors.

Material removal was calculated by using the Equation (1). The weight of the Ti6Al4V was measured before and after the machining of samples.

(1) $$\mathrm{MRR}=\frac{\Delta W\times 1000}{\rho \times t}$$

where Δ*W*, *ρ*, and *t* depicted the difference in weight after machining in grams, work density of Ti6Al4V in g/cm^3^, and machining time in seconds.

SR was examined with the use of an SJ-410 tester made by Mitutoyo (Mitutoyo Ltd., New-Dehi, India). Three different readings were taken for the average SR value, and its average was taken for analysis.

SEM was preferred to reveal the machined surface topography.

### 2.3. Optimization

The teaching–learning-based optimization (TLBO) method has been used in the present work. Rao and Patel [49] established the TLBO algorithm to solve multiobjective problems in various processes. TLBO operates on a teaching–learning methodology employed between a teacher and students. Students are considered as the population. The teacher teaches different subjects as constraints. The student with the highest marks in the class is regarded as the best learner. By adjusting the mean of a student’s marks during implementation, a teacher attempts to bring the results of the remaining students as closely as possible to the student who received the highest grades. The teacher phase of the TLBO algorithm includes teaching from the teacher, and the learner phase includes student interaction. In the teaching phase, the solution is updated to reflect the change in the present and the new mean *DM_j_* [50].

*DM_j_* = *r_j_* (*M_new_* − *T_F_ M_j_*)

*X_new,j_* = *X_old,j_* + *DM_j_*

$$T_{F}=Round\;\left(1+rand\left(0,1\right)\right)$$

*T_F_* is the teaching factor that decides the value of the mean to be changed. The value of *T_F_* can be either 1 or 2. The value of *T_F_* is decided randomly with equal probability. The value of *T_F_* is not given as an input to the algorithm and its value is randomly decided by the algorithm. The RI is a random number between 0 and 1, *M_j_* is the average score at iteration *j*, and *M_new_* is the new mean that the teacher obtained at iteration *j*. The second stage of the TLBO algorithm is the student phase. In the student phase, the solutions are improved by random interaction between the other solutions. To improve the current answer from *X_old_*_,*j*_ to *X_new_*_,*j*_, any two random solutions from the population, such as *X_j_* and *X_k_*, are first compared. The process is then carried out once more for the full population as follows:

If *f* (*X_j_*) < *f* (*X_k_*),

*X_new,j_* = *X_old,j_* + *r_j_* (*X_j_* − *X_k_*)

*Otherwise*

*X_new,j_* = *X_old,j_* + *r_j_* (*X_k_* − *X_j_*)

## 3. Results and Discussion

This section contains a comprehensive analysis of EDM parameters and their influence on MRR and SR measures. Firstly, the obtained results were analyzed through the statistical technique. The effect of individual factors was then studied on output measures. The TLBO algorithm was then used for the optimization of MRR and SR. Lastly, the effect of aluminum oxide (Al_2_O_3_) nanopowder and nano-graphene powder was studied on output measures.

Table 2 represents the experimental matrix created through Taguchi’s design and the obtained results of responses. All the experimental trials were repeated three times and their average value was considered during the analysis. Thus, the MRR and SR values represent the average values of three trials. It shows the maximum MRR of 10.6713 mm^3^/s for trial run 7, and the least SR of 4.35 µm for trial run 3.

The Minitab v17 software was utilized to generate the empirical regressions for output factors in terms of EDM parameters. The generated regressions play a key role in predicting the response values within the design matrix for any value of input factors. Regressions for MRR and SR were depicted in Equations (2) and (3), respectively.

(2) $$\mathrm{MRR}=8.218+0.0727\cdot T_{\mathrm{on}}-0.7506\cdot T_{\mathrm{off}}+0.1435\cdot \mathrm{Current}$$

(3) $$\mathrm{SR}\;=6.336+0.3500\cdot T_{\mathrm{on}}-0.2506\cdot T_{\mathrm{off}}-0.0123\cdot \mathrm{Current}$$

### 3.1. Analysis of MRR

**Figure 3:**
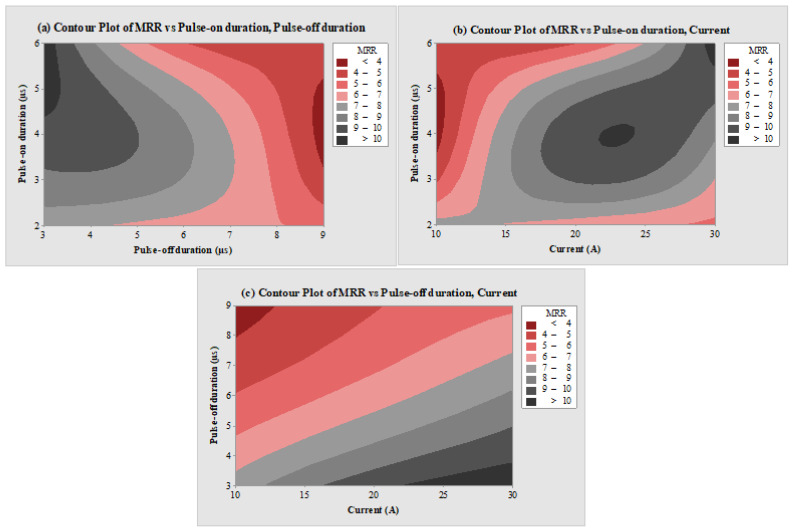
Counter plots of (**a**) MRR vs. T_on_ and T_off_, (**b**) MRR vs. T_on_ and current, and (**c**) MRR vs. T_off_ and current.

Figure 3a–c depicted the impact of EDM variables on the output response measure of MRR by using contour plots. In the contour plot, the third input process parameter was kept constant at the level 2 value. The plot of MRR vs. T_on_ and T_off_, as represented in Figure 3a, depicted that maximum MRR can be achieved at higher values of T_on_ and lower values of T_off_, while the lowest value can be observed at higher levels of T_off_. The main reason behind this is that an increase in T_on_ value enhances the spark duration, which in turn increases the rate of erosion owing to faster melting and vaporization of the work material [51]. Also, at higher levels of T_off,_ the sparking frequency gets reduced owing to the wider duration between the sparks. Thus, the thermal energy and discharge energy drop at lower values by reducing the rate of erosion [52]. Due to this reason, MRR was observed to be higher at the lower value of T_off_ and higher value of T_on_. Similar findings can be observed for the levels of T_on_ and T_off_ in Figure 3b,c. MRR was found to be maximum at the highest level of T_on_ in Figure 3b and the lowest level of T_off_ in Figure 3c. Figure 3b of MRR vs. T_on_ and current, and Figure 3c of MRR vs. T_off_ and current depict enhancement in MRR response at higher levels of current. This was due to the increased discharge energy. It further increases the thermal energy and enhances the sparking distribution, which melts and vaporizes more particles from the work material at a greater rate [53,54].

The results obtained in Table 2 as per Taguchi’s array were further analyzed using a statistical technique, called analysis of variance (ANOVA). During the regression study, 95% of CI has been considered. Under this, the *p*-value of the input variable should not be more than 0.05 to show the significant impact on the elected output response [55].

ANOVA results for MRR are represented in Table 3. The regression model term was observed to be significant, which shows that the generated regression is adequate. Among the input factors, T_off_ and current were found to have a vital role in the change of MRR response, while T_on_ was observed to be an insignificant factor. A higher F-value of 175.26 for T_off_ suggested that it has the largest significant effect, with a contribution of 69.51% trailed by the current with a 28.22% contribution. R-square values of the model suggest the adequacy and accuracy of the generated model if their value is near unity [56]. R-square values from Table 3 have demonstrated the suitability of the developed regression model.

### 3.2. Analysis of SR

**Figure 4:**
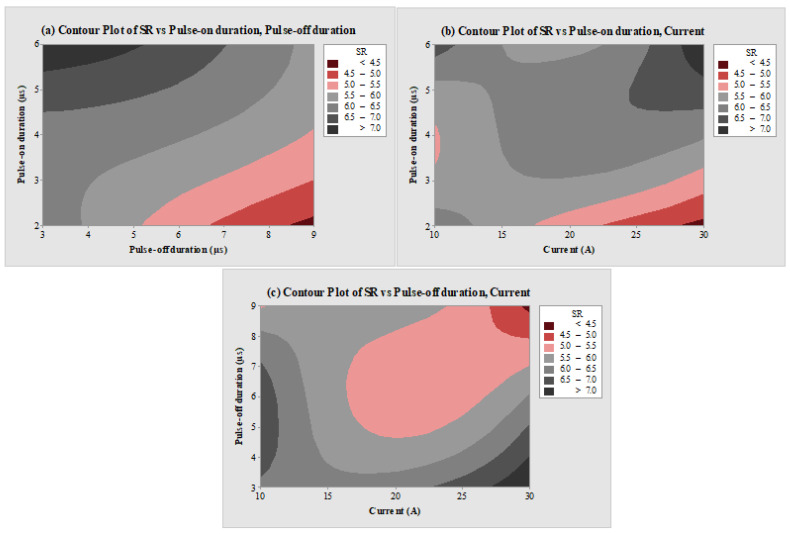
Counter plots of (**a**) SR vs. T_on_ and T_off_, (**b**) SR vs. T_on_ and current, and (**c**) SR vs. T_off_ and current.

The impact of EDM variables on SR is represented in Figure 4a–c through contour plots. In the contour plot, the third input process parameter was kept constant at the level 2 value. Figure 4a depicts the plot of MRR vs. T_on_ and T_off_. An increase in levels of T_on_ showed a negative effect on SR as the SR value was found to be higher, while the increased value of T_off_ has a reduced SR response. The lowest SR value (<4.5 µm) was observed at the highest levels of T_off_, while maximum SR (>7 µm) was found at the highest levels of T_on_. An increase in T_on_ value enhances the sparking frequency, thereby enhancing the rate of erosion. This created larger and deeper craters on the machined surfaces [57]. Thus, the SR value increases with the T_on_ value. With an increment in T_off_, due to less active sparks between the tool and workpiece, SR was observed to follow a downward path due to less thermal energy at the tool–work interface [54]. Similar findings can be observed for the levels of T_on_ and T_off_ in Figure 4b,c. SR was found to be maximum at the highest level of T_on_ in Figure 4b and at the lowest level of T_off_ in Figure 4c. Figure 4b of SR vs. T_on_ and current, and Figure 4c of MRR vs. T_off_ and current depict enhancement in SR response at higher levels of current. This was due to the increased discharge energy. It further increases the thermal energy and enhances the sparking distribution, which melts and vaporizes more particles from work material at a greater rate [58]. This in turn creates deeper and larger craters and thus enhances SR value [59].

Table 4 depicts the statistical outcomes of ANOVA for SR response. The regression model term was observed to be significant which shows that the ANOVA findings are suitable for the selected levels. T_on_ and T_off_ were detected as significant variables, while current was observed to be an insignificant factor. An f-value of 55.73 for T_off_ suggested that it has the largest significant impact, with a contribution of 50.41%, trailed by T_on_ with 43.71%. R-square values of the model suggest the adequacy and accuracy of the generated model if their value is near unity. R-square values from Table 4 demonstrate the fitness of the developed model.

### 3.3. Optimization

The conflicting conditions of input factors are evident from the statistical analysis of ANOVA and main effect plots for MRR and SR. This raises a need for an optimization strategy to be implemented. The TLBO algorithm was employed to obtain the best solutions for multiple output performance variables. TLBO method was used for multi- and single-objective optimization of MRR and SR. For the current investigation, the response of MRR was taken as the maximum criterion for increased machining efficiency and SR was marked as the minimum criterion for better surface quality. During the implementation of TLBO, upper and lower bounds of machine variables T_on_, T_off_, and current were selected between 2 µs to 6 µs, 3 µs to 9 µs, and 10 A to 30 A, respectively.

Individual output factors were optimized. The results are depicted in Table 5. A contradictory condition was observed between the response measure values in correspondence with the input factors. For the largest MRR condition, SR was also increased, which is undesirable. Similarly, the lowest SR values can be achieved, but subsequently, this also reduces the MRR value. So, the combination of parameters was conflicting, and this shows that a single-objective optimization can be used to maximize and minimize either parameter. This can be solved by employing a multiobjective optimization method.

The multiobjective TLBO process was adopted for the simultaneous optimum values of MRR, and SR response measures. MOTLBO generates nondominant solutions for input variables, and can manage two or more output responses. Table 6 displays the results of Pareto points, consisting of values of response measures and the input factors of the EDM process. Each Pareto point shows the unique optimal outcome. As per the specific need of response values, the user can select the appropriate input conditions to fulfill the required condition. All these results were validated through experimental trials. A minimal error of less than 5% was observed between experimental and predicted results, concluding the acceptability of regression models with the TLBO technique. Thus, it demonstrates the viability of the created regressions and the TLBO method for the EDM process.

### 3.4. Investigating the Effect of Alumina and Nano-Graphene Powders on MRR and SR

The influence of aluminum oxide (Al_2_O_3_) nanopowder and nano-graphene particles were investigated on SR, MRR, and surface morphology. Nanopowders were used at 1 g/L amount in the dielectric fluid. For the analysis, an objective function with an equal weightage of 0.5 was assigned to output measures.

(4) $$Obj\;\left(v_{1}\right)=w_{1}\cdot \left(MRR\right)+w_{2}\cdot \left(SR\right)$$

This simultaneous optimization yielded an MRR of 8.9154 mm^3^/s and an SR of 5.14 µm for conventional EDM. The objective function shown in Equation (4) has input factors at T_on_ of 2 µs, T_off_ of 5 µs, and current of 30 A. To validate the results, an experimental trial was conducted at input factor levels. The validation trial showed an MRR of 8.9811 mm^3^/min and an SR of 5.05 µm. Thus, an error of less than 5% was observed between experimental and predicted results, concluding the acceptability of regression models with the TLBO technique. Another trial was conducted by using alumina and graphene powders at 1 g/L to compare the results with the conventional EDM process. Table 7 depicts the obtained results. It can be observed that MRR and SR values were improved by using both nanopowders. This was because the addition of nanopowders enlarged the thermal conductivity of the dielectric, increased the discharge gap, decreased the breakdown strength, and enhanced the spark difference [60,61,62]. It also facilitated the proper flushing of eroded particles [63]. Thus, the machining performance was significantly improved by using powder-mixed EDM as compared to conventional EDM. For alumina powder, the performance of MRR and SR was improved by 35.19%, and 18.27%, respectively. In the case of nano-graphene powder, MRR and SR showed a larger improvement of 45.81%, and 37.22%, respectively. The reason behind the larger improvement with the use of nano-graphene powder was the higher thermal conductivity as compared to the alumina powder [64,65]. Increased thermal conductivity lowers the breakdown strength of the dielectric fluid and intensifies the discharge gap [66,67].

### 3.5. Investigating the Effect of Alumina and Nano-Graphene Powders on Machined Surfaces

**Figure 5:**
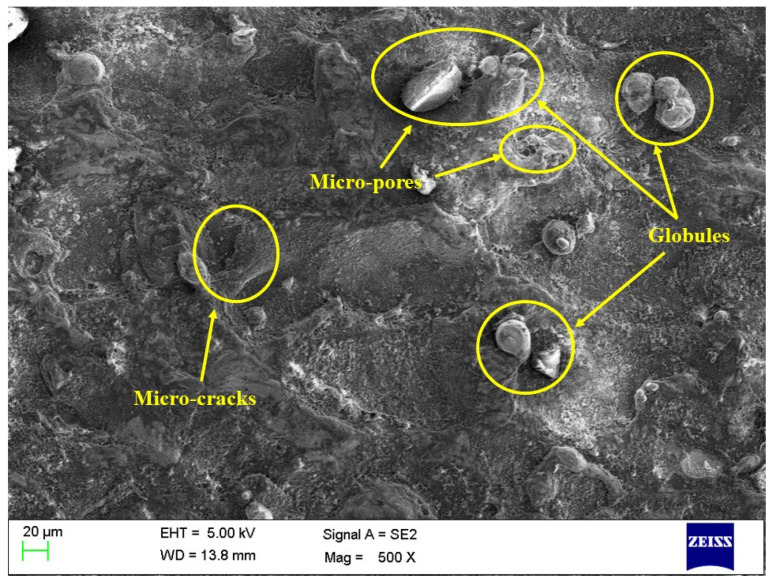
Surface morphology for conventional PEDM.

**Figure 6:**
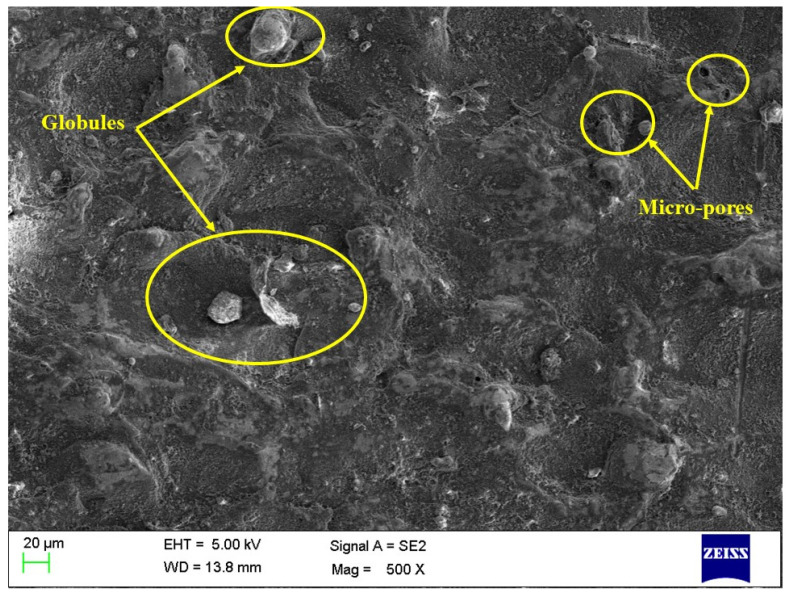
Surface morphology for PMEDM using alumina nanopowder.

**Figure 7:**
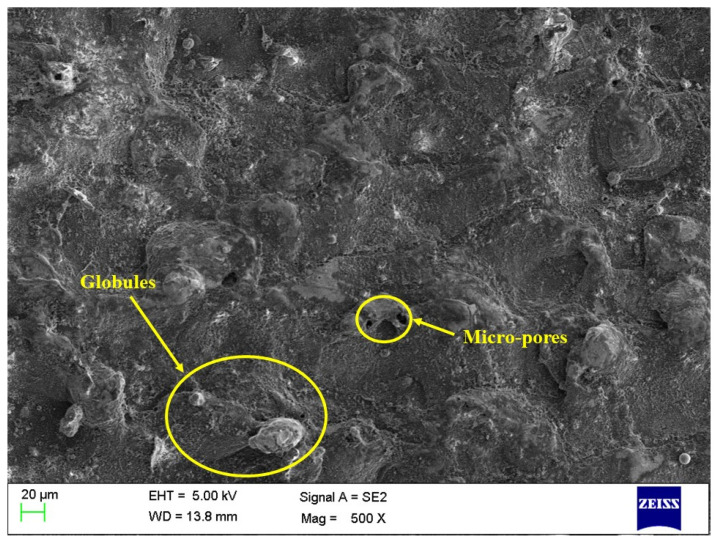
Surface morphology for PMEDM using nano-graphene powder.

The influence of aluminum oxide (Al_2_O_3_) nanopowder and nano-graphene powder was investigated on the surface morphology of machined surfaces. The EDM process has a set of parameters that must be controlled carefully to obtain a machined surface free of defects like microcracks, pores, and globules. The results shown in Table 7 for PMEDM processes show the higher significance of the PMEDM process in comparison with conventional EDM. However, it also becomes essential to evaluate the surface defects on the machined components. Thus, Figure 5, Figure 6 and Figure 7 depict the SEM images of the machined surface for conventional EDM, PMEDM with alumina powder, and PMEDM with nano-graphene powder, respectively. The machined surface obtained for conventional EDM depicted more surface defects than the PMEDM process. The surface morphology of PMEDM using nano-graphene showed the fewest surface defects in terms of microcracks, pores, and globule size. The higher conductivity of nanopowder stabilized the machining process and widened the machining gap with improved flushing of debris [68,69,70]. Due to this reason, it showed improved machined surfaces.

## 4. Conclusions

The present study investigated the effect of two different nanopowders, namely alumina and nano-graphene, to analyze their effect on MRR, SR, and surface morphology. The experimental runs were performed by using Taguchi’s design with T_on_, *I*, and T_off_ as input factors. The following conclusions were drawn from the obtained results:

➢ Empirical relations were generated through Minitab. ANOVA was employed to study the statistical significance. The regression model term was observed to be significant for both SR and MRR responses.
➢ Among the input factors, T_off_ and current were found to have a vital role in the change of MRR response. A higher F-value of 175.26 for T_off_ suggested that it has the largest significant effect, with a contribution of 69.51%, trailed by current with a 28.22% contribution. For SR response, T_on_ and T_off_ were identified as significant factors. A higher F-value of 55.73 for T_off_ suggested that it has the largest significant impact, with a contribution of 50.41%, trailed by T_on_ with 43.71%.
➢ Single-objective optimization has shown a maximum MRR of 10.7071 mm^3^/s and a least SR of 4.41 µm. The objective function of simultaneous optimization has given an optimum MRR of 8.9154 mm^3^/s and an SR of 5.14 µm at input factors of T_on_ at 2 µs, T_off_ at 5 µs, and *I* at 30 A.
➢ The influence of alumina and nano-graphene powder was investigated on MRR, SR, and surface morphology at optimized parametric settings. The machining performance was significantly improved by using both powder-mixed EDM as compared to conventional EDM. For alumina powder, the performance of MRR and SR was improved by 35.19% and 18.27%, respectively. In the case of nano-graphene powder, MRR and SR showed a larger improvement of 45.81% and 37.22%, respectively. Due to the higher conductivity of nano-graphene powder, it showed a larger improvement as compared to alumina powder.
➢ Lastly, SEM was utilized to investigate the impact of alumina and graphene powder on surface morphology. The machined surface obtained for conventional EDM depicted more surface defects than the PMEDM process. The surface morphology of PMEDM using nano-graphene showed the fewest surface defects in terms of microcracks, pores, and globule size.
➢ The last thing to say is that workers who deal with nanoparticles in dielectrics must wear gloves and masks to avoid the risk of skin exposure. Exposure can occur during pouring or mixing operations; the use in EDM will need further research.

## Figures and Tables

**Table 1:** Experimental conditions.

Machining Factors	Levels/Values
Pulse-on duration (µs)	1, 3, 5
Pulse-off time (µs)	6, 16, 26
Current (A)	20, 60, 100
Cutting depth	2 mm
Spark gap	0.01 mm
Nanopowder	Al_2_O_3_, and nano-graphene

**Table 2:** Evaluation of MRR, and SR versus EDM factors.

Run Order	T_on_ (µs)	T_off_ (µs)	Current (A)	MRR Trial 1	MRR Trial 2	MRR Trial 3	Avg. MRR (mm^3^/s)	SR Trial 1	SR Trial 2	SR Trial 3	Avg. SR (µm)
1	2	3	10	7.4297	7.5738	7.2601	7.4212	6.63	6.12	6.34	6.36
2	2	6	20	6.5181	6.5752	6.5501	6.5478	5.09	5.24	5.31	5.21
3	2	9	30	5.7386	5.8801	5.8105	5.8097	4.28	4.29	4.47	4.35
4	4	3	20	9.8459	9.7601	9.5341	9.7134	6.26	6.43	6.27	6.32
5	4	6	30	8.1933	8.2927	8.0167	8.1677	6.16	5.89	6.13	6.06
6	4	9	10	3.6601	3.4211	3.6021	3.5611	5.38	5.48	5.49	5.45
7	6	3	30	10.9398	10.1099	10.9642	10.6713	7.33	7.67	7.41	7.47
8	6	6	10	5.0007	5.0607	5.1073	5.0562	6.73	6.75	6.94	6.81
9	6	9	20	4.8894	4.9702	4.9106	4.9234	5.96	5.6	5.97	5.84

**Table 3:** Statistical analysis for MRR.

Source	Adj. SS	F	*p*	% Contribution
Regression	42.9104	82.39	0.000	Significant
T_on_	0.1268	0.73	0.432	Insignificant
T_off_	30.4277	175.26	0.000	Significant
Current	12.3559	71.17	0.000	Significant
Error	0.8681			
Total	43.7784			

R^2^ = 98.02%, R^2^ adj. = 96.83%, R^2^ pred. = 94.57%.

**Table 4:** Statistical analysis for SR.

Source	Adj. SS	F	*p*	Significance
Regression	6.4212	35.19	0.001	Significant
T_on_	2.9400	48.33	0.001	Significant
T_off_	3.3900	55.73	0.001	Significant
Current	0.0912	1.50	0.275	Insignificant
Error	0.3041			
Total	6.7254			

R^2^ = 95.48%, R^2^ adj. = 92.76%, R^2^ pred. = 86.25%.

**Table 5:** Single-objective optimization.

Condition	EDM Variables			Output Response	
	T_off_ (µs)	T_off_ (µs)	Current (A)	MRR (mm^3^/s)	SR (µm)
Maximum MRR	6	3	30	10.7074	7.32
Minimum SR	2	9	30	5.9130	4.41

**Table 6:** Pareto optimal points.

Sr. No.	T_on_ (µs)	T_off_ (µs)	Current (A)	MRR (mm^3^/s)	SR (µm)
1	6	3	30	10.7074	7.32
2	5	3	30	10.6347	6.97
3	4	3	30	10.5620	6.62
4	3	3	30	10.4893	6.27
5	2	3	30	10.4166	5.92
6	2	4	30	9.6660	5.66
7	2	5	30	8.9154	5.41
8	2	6	30	8.1648	5.16
9	2	7	30	7.4142	4.91
10	2	8	30	6.6636	4.66
11	2	9	30	5.9130	4.41

**Table 7:** Effect of alumina and graphene powders on MRR and SR.

Experimental Condition	Input Factors	Output Responses
Conventional EDM	T_on_ = 2 µs T_off_ = 5 µs Current = 30 A	MRR = 8.9811 mm^3^/s SR = 5.05 µm
Aluminum oxide (Al_2_O_3_) nanopowder	T_on_ = 2 µs T_off_ = 5 µs Current = 30 A Alumina nanopowder = 1 g/L	MRR = 13.8568 mm^3^/s SR = 4.27 µm
Nano-graphene powder	T_on_ = 2 µs T_off_ = 5 µs Current = 30 A Nano-graphene powder = 1 g/L	MRR = 16.5732 mm^3^/s SR = 3.68 µm

## Data Availability

Data are contained within the article.
